# How to communicate with older adults about climate change: a systematic review

**DOI:** 10.3389/fpubh.2024.1347935

**Published:** 2024-04-04

**Authors:** Samuele Pinna, Diego Longo, Patrizio Zanobini, Chiara Lorini, Guglielmo Bonaccorsi, Marco Baccini, Francesca Cecchi

**Affiliations:** ^1^Department of Experimental and Clinical Medicine, University of Florence, Florence, Italy; ^2^Fondazione don Carlo Gnocchi, Scientific Institute, Florence, Italy; ^3^Department of Health Science, University of Florence, Florence, Italy

**Keywords:** communication, climate change, older adults, awareness, engagement, behavioral change

## Abstract

**Introduction:**

Although older adults are particularly vulnerable to the effects of climate change, they seem to be overall less concerned about it, and less inclined to support climate policies. The study aims to identify the communication strategies that have been evaluated in promoting awareness and/or climate friendly behaviors in older adults.

**Methods:**

We searched multiple electronic databases for studies that evaluated the effects of any interventions aimed at communicating climate change to older persons (over 65 years) and assessed the results as awareness and /or behavioral changes. We selected quantitative, qualitative and mixed methods studies, and we also included systematic reviews for cross-referencing. Risk of bias of included studies was evaluated using different tools according to the study design.

**Results:**

From a total of 5,486 articles, only 3 studies were included. One mixed-method study engaged older adults to assess the community vulnerability to climate change and to develop adaptation recommendations based on their perspectives; one qualitative study conducted focus groups to identify the more effective language, values and themes based on participants’ responses to narratives; one quantitative study utilized a 360-degree audio-visual platform allowing users to engage with immersive visualizations of sea-level rise scenarios.

**Discussion:**

Despite the paucity of literature, this review demonstrates the potential for different strategies to increase the awareness of older persons about climate change. The involvement of older adults in the communication process, the identification of their priorities, and the integration of technology in their daily lives are promising approaches but more research, including both quantitative and qualitative studies is recommended on this topic.

**Systematic review registeration:**

For further details about the protocol, this systematic review has been registered on PROSPERO on July 1, 2023 (https://www.crd.york.ac.uk/prospero/display_record.php?ID=CRD42023438256).

## 1 Introduction

The effects of climate change are pervasive: they impact the food, air, water, and shelter, and affect every region of the world and every income and age group (1). Changing climate has produced considerable effects in the social and environmental determinants of health level (1), such as an increase in morbidity and mortality due to heat stress and heatstroke (2, 3). Changes in many extreme weather and climate events have been observed since about 1950. Some of these changes, including a decrease in cold temperature extremes and an increase in warm temperature extremes, extreme high sea levels and number of heavy precipitation events in several regions, have been linked to human influences (1). Increasing trends in extreme precipitation and discharges in some catchments implies greater risks of flooding on a regional scale. However, the character and severity of impacts from climate change and extreme events emerge from both climate-related hazards and vulnerability of human and natural systems (1). For example, the increasing costs related to flood damage are partly due to the increasing exposure of people and assets (1).

The older adult population represent a vulnerable group that is particularly susceptible to the effects of climate change due to factors such as age-related physiological changes, pre-existing health conditions, and limited mobility and adaptive capacity (4, 5). Those at higher risk are persons older than 65 years, those with disabilities or pre-existing medical conditions, those working outdoors or in non-cooled environments, and those living in regions already at the limits for human habitation (6).

Although there is no agreed common definition of older persons, most agree on confining such definition to persons aged 65 years and older (7). This age group is the fastest growing worldwide (8). Two-thirds of the world’s older adults today live in emerging countries and most countries are experiencing growth in the number and proportion of older persons in their population (9).

Various elements such as the natural physiological ageing process, physical and cognitive limitations, as well as socioeconomic factors, shape how older individuals react to potentially challenging environmental shifts. This combination of influences, coupled with underlying health issues, heightens their susceptibility to significant emergencies (10).

The need to find out how to engage older people with climate change is increasingly being recognised (9, 11–14). Actually, although the relationship between age and climate change denial is not fully clear, there is fairly consistent evidence that younger adults are more concerned about climate change than are older individuals (15–18), who seem to be overall less worried, less likely to allocate public funds to environmental purposes, and less inclined to support climate policies (4).

Climate change communication to older persons is a crucial aspect to better develop future lines of research in this field. This demographic group is considered one of the most vulnerable to health effects of climate change (19), thus making the role of communication critical in highlighting the resulting risks and safeguarding their well-being (20). In addition, older persons represent a large segment of the population with opinions and behaviors that are often relevant in societal debates that can lead to significant changes in society itself (21). As leaders in their communities and repositories of traditional knowledge, older individuals can serve as resilient models for climate change adaptation and mitigation efforts, particularly in low-resource environments where their experiential wisdom is invaluable (22), Raising awareness of the issue through the communication process could therefore play a key role in these processes.

Communicating climate change to the older adults can also have an important impact on future generations. These are, in fact, repositories of lived experience that could help connect current climate events to their past experiences by demonstrating how the climate has changed over time. From this perspective, developing an awareness of having been actors in this change, through their own daily actions and lifestyles, could also lead to behavioral change and greater involvement in order to legacy a more sustainable future for future generations (23).

Existing research suggests several major barriers that may depress the interest of older persons in environmental action and limit their opportunities to engage in it. Older persons often report a lack of sufficient expertise or knowledge about environmental issues and science in general to contribute effectively, that can depress their self-efficacy and participation (3) to climate action. The age gap that still persists in using information technology also inhibits older participation in some environmental activities, which may rely on the internet for recruitment and engagement (14).

Globally, targeting environmental action to older persons provides an excellent opportunity to address two pressing social problems simultaneously: the need for greater social integration and participation of older persons and the mounting concern about the sustainability of the natural environment result (14). Environmental action in the older population can be life-enhancing to the individuals and beneficial to their communities, but this has received limited research attention yet, especially in low medium income countries (14).

Communication, information and education on climate change issues are considered important to mobilize people and catalyze action. Informing people and conveying messages on climate change is a difficult task; in addition to knowledge about the theoretical aspects of communication, it is important to strike the right balance in conveying the right messages and tailoring communication the target population needs (24). Describing climate change as an emergency or crisis, for example, has become increasingly common, and although research indicates that many people see climate change as urgent (25), how people react to this language can vary widely from feeling threatened to feeling energized (26).

Pro-environmental communication is defined as a tool to educate people and inform their behavior in order to reach a more sustainable lifestyle (27). This behavior-centred approach to climate change is crucial, since the technology is not sufficient in itself to slow the environmental crisis. However, behavior is influenced both by subjective (motivation, abilities) and objective (context, barriers to environmental action) conditions, thus any effective communication strategy should consider both (27).

There has been very little research about how to communicate with older persons about climate change (28). Older persons are relatively invisible in climate discussions compared to the youth demographic, yet they are arguably the most critical for broader climate action.

While today’s youth may be exposed to climate change education in school, the older adults were not, and little climate change communication to date has been targeted at older audiences.

Thus, understanding the strategies in the context of climate change communication targeting older persons is critical to designing tailored interventions and initiatives that promote action against the consequences of climate change.

The purpose of this systematic review is to identify the communication strategies that have been evaluated in promoting awareness and/or also climate friendly behaviors in older adults.

## 2 Methods

This review followed the guidelines of the Preferred Reporting Items for Systematic Reviews and Meta-Analyses (29) and was registered in PROSPERO on 01 July 2023.

In this review we searched for studies published in English that provided an intervention aimed at communicating climate change information to older people than 65 years of age and assessed its effectiveness in terms of awareness and/or behavioral changes using any method (questionnaires, tracking systems, observation) (Table 1).

**Table 1:** The PICO scheme of the review.

P - population	Older Persons over 65
I - intervention	All kinds of intervention
C - comparison/control	Any control or no control
O - outcome	Increase of awareness and/or behavioral changes in relation to climate change

### 2.1 Search methods for identification of studies

We searched articles in the following electronic databases from inception to April 26, 2023: PsycInfo, EBSCO Edu Source, EBSCO, Green File, EMBASE, PubMed, WoS, CINHAL, Scopus. We followed the search strategy in keeping with the PICO scheme—Cochrane Handbook for Systematic Reviews of Interventions (30) which is summarized in the following question: “Are intervention focused on communication strategies useful in increasing awareness and/or behavioral changes in relation to climate changes in older persons? (Table 1). It was developed in consultation with a research group experienced in delivering health communication and health literacy to older persons.

First, we conducted an exploratory search on PubMed, Google Scholar, and Google Search with the aim of finding essential papers. Then, we utilised the retrieved articles and a thesaurus software (Power thesaurus) to identify keywords and synonyms reflecting the concepts of climate change and interventions aimed at communication of climate change issues to the older population. Second, we constructed the search string in accordance with the PICO scheme.

**Figure 1:**
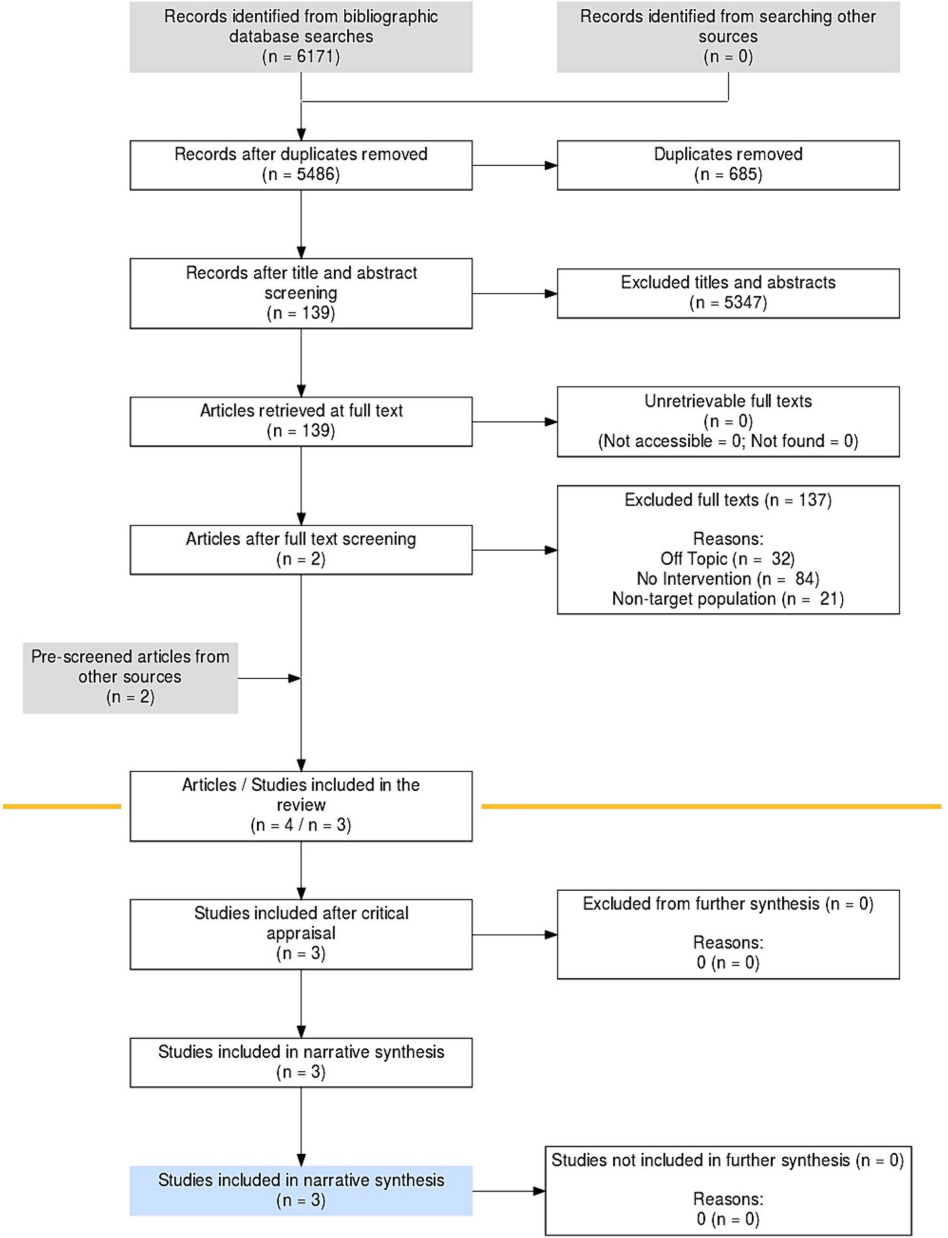
Flow diagram of the process of study selection.

To ensure retrieval of all eligible articles, reference lists of articles retrieved were evaluated for relevant publications. The guidance provided by the Preferred Reporting Items for Systematic Reviews and Meta-Analyses (PRISMA) flow diagram was employed to present studies identified through the database search that satisfied the inclusion and exclusion criteria (29) (Figure 1). The search in the electronic databases was conducted with the search filters in the Supplementary material.

### 2.2 Types of studies

No restrictions were made about the type of the studies delivered. We selected quantitative studies (randomized controlled trials, non-randomized controlled trials, before-after studies, single-subjects studies, cross-sectional studies, case–control studies, cohort studies, case series, case reports, field experiments, surveys), qualitative studies (phenomenological studies, surveys, focus groups) and mixed methods studies.

We also included systematic reviews and meta-analyses on this topic in order to possibly retrieve relevant articles by cross-referencing.

### 2.3 Population

We included studies where the target population was older persons, defined as adults aged 65 years or more. Studies that declared the enrolment of older people but used a lower age cutoff were excluded, unless they enrolled participants who were mostly older than 65 years of age. Studies that enrolled participants of various ages but reported separate analyses in a subgroup of participants who were older than 65 years of age were also included.

### 2.4 Intervention

All kinds of intervention focused on communication strategies to address climate change to older persons were included, including focus groups, educational approaches, meetings. We did not include studies that focused on improvement of health literacy, climate change literacy or decision-making processes. We also did not include studies concerning educational interventions in a school/university context, unless they were targeting older people.

### 2.5 Comparison

No restrictions were made about the comparator, when present. Thus, we included studies that compared an intervention either with no intervention, or with another active intervention or with a sham intervention.

### 2.6 Outcome

We included studies that measured the outcome as an increase of awareness and/or behavioral changes in relation to climate changes. The latter assesses the adoption of climate-friendly behaviors and actions by the older participants. The outcome could be measured through self-reported surveys, tracking systems, or observational data.

### 2.7 Data collections and analysis

#### 2.7.1 Study selection

One author (MB) screened the articles identified in the search to eliminate duplicates and obtain the title/abstract list of retrieved studies. Using this list, two authors (SP and DL) independently completed the title/abstract screening according to the eligibility criteria. Disagreements were solved by consensus with the participation of a third reviewer (FC). Then, we obtained the full texts of all eligible articles and two reviewers (SP and DL) independently completed the second stage of the study selection process to finally decide on their inclusion or exclusion. Conflicts in this phase were managed as aforementioned. Rayyan software (31) was used in every step of this selection process.

#### 2.7.2 Assessment of methodological quality

Two authors (DL and SP) independently evaluated the methodological quality of eligible studies. During this evaluation, the review authors were blinded to the source of publication and results. We planned to assess the methodological quality of included studies using the following tools: the Cochrane Risk of Bias Tool for randomized clinical trials; the Methodological Index for Non-Randomized Studies (MINORS) for non-randomized studies, both comparative and non-comparative; the Joanna Briggs Institute (JBI) Critical Appraisal Checklist for analytical cross-sectional studies, case reports and case series; the CASP Qualitative Studies Checklist for qualitative research; the Mixed-Methods Appraisal Tool (MMAT), version 2018, for mixed-methods studies; the AMSTAR (A MeaSurement Tool to Assess systematic Reviews) 2, for systematic reviews. Quality assessment has been conducted independently by two researchers. Any disagreements have been resolved by a third researcher.

### 2.8 Data extraction

Two review authors (SP and DL) independently recorded the following information using a self-developed form. This form reported information about:

- study design: quantitative (type), qualitative (type), mixed method (type);
- characteristics of the samples: number of participants, age, gender, level of education, occupation, ethnicity, social-economic conditions, living together, disability, geographical area (country, urban/rural zone);
- details of interventions (including control, when present): type (lectures, meetings, focus groups, provision of educational materials, movies. Etc.), delivery method (in-person or online, mixed), duration and number of each intervention, overall duration;
- outcome measures: type of outcome measured (awareness, behavioral changes or both), method of measuring (self-reported surveys, tracking systems, or observational data) and timing of measurements;
- data relevant to the evaluation of methodological quality, as required by the specific appraisal tool used.

### 2.9 Data analysis

For controlled trials we planned to compute different effect measures, both as point estimates and the 95% Confidence Interval (95%CI), for continuous and dichotomous variables. When the outcome is expressed as continuous data, we would compute the mean difference (MD, i.e., the absolute difference between the mean value in the two groups) or the standardized mean difference (SMD, i.e., the difference between the mean value in the two groups divided by the standard deviation among participants) when the pooled trials used the same rating scale/test and when they used different rating scales/tests for the same domain, respectively.

When the outcome was expressed as dichotomous data, we planned to compute the risk ratio (RR, i.e., the ratio of the risk of the event in the two groups) and odds ratio (OR), (i.e., the ratio of the odds of the event in the two groups). Large ordinal variables would be analyzed as continuous variables and shorter ordinal variables would be transformed into dichotomous by grouping adjacent categories. When possible, we also planned to pool the results of trials to obtain an overall estimate of the treatment effects, testing heterogeneity and inconsistency among trials using the I2 statistic and the Q statistic, respectively. We would use either random-effects or fixed-effects models to estimate pooled effects, depending on the presence or absence of substantial (I^2^ above 50%) heterogeneity. For the other types of studies, a narrative analysis is planned.

## 3 Results

### 3.1 Results of the search

This search yielded a total 6,171 before removing duplicates, including 600 on PUBMED, 108 on EBSCOhost Green File, 156 on EBSCOhost EduSource, 74 on CINHAL, 99 on PsycInfo, 538 on EMBASE, 2815 on Scopus, 1781 on WoS. Removal of duplicates resulted in 5486 titles that were screened for inclusion. After screening titles and abstracts, 114 full texts were assessed for eligibility and 2 studies (28, 32) were identified that met the inclusion criteria. One additional article (33) was included from the reference list of Rhoades et al. (29), closely related to it since it reported data from the first phase of the same research. One further article (31) was retrieved from the reference list of Latter (25). Ultimately, therefore, 4 articles reporting data from 3 studies were included. The flowchart diagram of the entire selection process is shown in Figure 1.

### 3.2 Included articles

The first study (32) that was selected from the search on electronic databases is a mixed-methods case study that examined the effects of a participatory adaptation planning process called “Climate Resilient Seniors” conducted on 37 older adults in the community of Bridgeport, Connecticut. This study is strictly connected to another paper from the same group and with the same study design (33). Given the close correlation between the two articles, we decided to analyse the methods and results of both as two parts of a same research project, as stated by the authors themselves. The project follows an adaptation planning model consisting of four steps, the first two of which are described in the first article and the others in the second article.

The first part (33) was aimed at identifying current and predicted future climate changes relevant to the older adults’ community of Bridgeport and assessing the resulting vulnerabilities and risk to the community. It consisted of two participatory meetings open to all Bridgeport seniors, attended by 55 individuals. In these meetings, participants engaged in a discussion-based process to consider the impact on their lives of current and predicted climate changes, the factors that contributed to their vulnerability and the strategies they adopted to deal with the problem. The aim of this process of engagement was to assess the community vulnerability to climate change in terms of personal characteristics and contextual factors. Although this goal may seem inconsistent with the criteria used to select relevant articles in this systematic review, we decided to include also this article, pointing out that it must be considered with the second part as a unique study, because the project is the same, and we think the adopted methodology in the first part was critical to the achievement of the overall results of the project.

A 5-points Likert scale survey to rank the participants’ level of concern resulted from this phase. The 31 factors included in the survey were based on the discussions during the two meetings and supplemented by issues raised in the scientific literature. Subsequently, according to the participants’ recommendation and in an effort to reach a diverse representation of Bridgeport’s older adults’ community, the survey was distributed through organizations that engaged with it. One hundred sixty-four older adults joined the survey and indicated diverse concerns over climate related stressors with all 31 questions ranked as a high concern (a four or five on the 5-point Likert scale) by over 15% of respondents.

The most pressing concerns emerging from this survey were associated with receiving adequate warning, securing safe shelter, as well as getting assistance during and after climate related stressors.

In the second part of the project (33), two adaptation planning meetings were conducted in order to develop adaptation recommendations based on participants’ perspectives. Thirty-five older adults attended these meetings. This process started, in the first meeting, with the individuation of six adaptation goals in order to address the concerns raised in the vulnerability survey. To match these goals, a list of seven adaptation recommendations was developed. In the second meeting participants were asked to elicit additional feedback and prioritize the recommendations in terms of potential impact on reducing risk as well as potential feasibility of implementation. These two variables were ranked separately on a 5-points Likert scale. Those actions that received the highest score in both impact and feasibility were considered of highest priority. Finally, a summative evaluation assessed the outcomes of the whole project in terms of enhancing older adults’ resilience to climate change. This evaluation utilized a combination of participant observation, semi-structured focus group interviews and Likert scale surveys, and semi-structured interviews with attending city staff (n = 7).

Authors summarized the participants’ level of agreement to the 5 items selected for the summative evaluation in terms of average using a 5-points Likert scale, where 1 and 5 indicated strong disagreement and strong agreement, respectively. Results showed unanimous agreement among participants on the generalizability to older persons of the recommendations in terms of needs, concerns and community safety perception. Additional benefits of the project included raising awareness of climate related risks, increasing participants’ knowledge about protective actions and enhancing older adults’ ability to self-advocate.

Moreover, the inclusion of older persons in such a program led to an upholding of their dignity in terms of valuation of older people as stakeholders with expert knowledge about their vulnerability to climate change and as key contributors in understanding the effective measures to enhance their resilience.

The second article retrieved from the original search (28) is a qualitative study that used the Climate Outreach’s Narrative Workshop methodology (34)? to identify language, values and themes that work best in communicating with older people about climate change. This methodology looks at values and narratives to find effective ways to communicate climate change with specific audiences, creating a “discursive and conceptual space” where people share their own subjective understandings and reflections (35). This approach recognizes that engagement is subjective and is specifically concerned with the subjective views and behaviors of participants, taking an “insider view” rather than imposing specific meanings onto them (36). The authors conducted three 90-min in-person focus groups involving a total of 17 older adults in three English counties, in order to have a mix of urban and rural participants from different areas of England. After an initial phase of free discussion among participants without any explicit solicitation on the topic of climate change, 14 “narratives” (short texts) on the topic were presented for discussion. The narratives were written based on values, language and framing in relation to climate change that had been highlighted by previous research on the topic. For each narrative, participants were asked to highlight the words they felt as positive or negative, and then a brief group discussion was held about their decisions.

From the participants’ responses, the authors identified which narratives elicited a positive or negative feedback and resonated (or not) with them. The subsequent analysis, which included also the initial part of the focus groups, found that four key themes emerged, i.e., consideration and responsibility about climate change, community (including volunteering and connections with others), power (including governments and big organizations actions but also the power of individual actions) and international aspects of climate change (including global cooperation). A key finding from this study is that it is important to understand the core values of the older generation, as this affects how climate change issues should be communicated to be more likely to resonate with them.

Even with the limitations of a qualitative study that, by itself, does not analyse quantitatively participants’ responses, the authors of this article reported that participation in the focus groups, the opportunity to engage with their peers, and the choice of topics that were most meaningful to them, all contributed to an increase in their awareness of climate change and its effects, as revealed by the qualitative analysis, in coherence with our PICO criteria.

The third study (37) reports the results of an innovative approach to address climate change concerns among a mixed-age group of Marin County (CA) residents. The project, named Here-Now-US, aimed to test a novel visualization technology called “OWL” designed for sea-level rise scenarios. This technology utilized a 360-degree audio-visual platform, allowing users to engage with immersive visualizations, answer survey questions, and provide audio comments.

Placed in a residential area, the research team recorded over 3,700 viewing sessions in a 14-week timelapse. Participants were asked to answer a Likert-scale embedded survey about their levels of concern before and after the viewing experience. Other questions focused on their willingness to learn more about the topic or to be engaged in local adaptation planning efforts. Age was requested as well. The sample included: Gen Z (under 15 years), 21%; Millennials (18–35 years old), 18%; Gen X (36–50 years old), 26%; Baby Boomers (51–72 years old), 26%; Matures (more than 72 years old), 10%. The study was included in the present review because separate data from each age groups are presented in a related publication (17), available only online Here Now Us Project and Research Summary.pdf.^1^

Results from the study indicated that the OWL-based 3D visualizations effectively raised concerns about flood risks among users. By localizing sea-level rise to specific areas where impacts are expected, the visualizations increased awareness and understanding of localized climate change risks. Notably, the visualizations were particularly effective among those initially showing low or no concern about current flooding risks. This subgroup exhibited an average shift of two concern levels after viewing the sea-level rise visual.

The study also explored age-related differences among OWL users, and found that older age groups, particularly Matures and Baby Boomers, showed the highest levels of concern about existing flooding risks. When presented with the sea-level rise scenario, these groups demonstrated the most significant shifts toward greater concern, indicating a positive correlation between age and concern levels. In detail, in the oldest age group the prevalence of people stating that they were “not at all” or “not very” concerned fell from 39 to 31 percent, and that of people stating that they were “very” or “extremely” concerned rose from 40 to 47 percent. Further analysis revealed that the oldest and youngest age groups expressed the greatest interest in community engagement, with a statistically significant relationship between age and the desired level of engagement. Twenty-five percent of the older group, compared with 6–8% of the other groups, said they were willing to take an active role in the community on this issue. In conclusion, the OWL technology proved highly effective in raising concern and motivating individuals, especially older Americans, to become actively involved in community adaptation planning. The results underscore the importance of tailoring outreach efforts to older age groups who show a willingness to contribute to climate action initiatives and leave a positive impact on future generations.

### 3.3 Quality appraisal

The included studies were all of moderate/high quality, in details, the results of the quality evaluation are shown in Table 2 [MMAT for the study reported in two separate publications (29, 30); Table 3] [MINORS checklist for the study reported by Moser (31) and Table 4] [CASP checklist for qualitative researches for the study reported by Latter (25)].

**Table 2:** Quality appraisal (Mixed-Methods Appraisal Tool) of the mixed-method study included in the review (29, 30).

	Yes	No	?
*Screening questions (all types)*			
S1. Are there clear research questions?	x		
S2. Do the collected data allow to address the research questions?	x		
*1. Qualitative studies*			
1.1. Is the qualitative approach appropriate to answer the research question?	x		
1.2. Are the qualitative data collection methods adequate to address the research question?	x		
1.3. Are the findings adequately derived from the data?	x		
1.4. Is the interpretation of results sufficiently substantiated by data?	x		
1.5. Is there coherence between qualitative data sources, collection, analysis and interpretation?	x		
*4. Quantitative descriptive studies*			
4.1. Is the sampling strategy relevant to address the research question?	x		
4.2. Is the sample representative of the target population?*		x	
4.3. Are the measurements appropriate?	x		
4.4. Is the risk of nonresponse bias low?	x		
4.5. Is the statistical analysis appropriate to answer the research question?**		x	
*5. Mixed-method studies*			
5.1. Is there an adequate rationale for using a mixed methods design to address the research question?	x		
5.2. Are the different components of the study effectively integrated to answer the research question?	x		
5.3. Are the outputs of the integration of qualitative and quantitative components adequately interpreted?	x		
5.4. Are divergences and inconsistencies between quantitative and qualitative results adequately addressed?	x		
5.5. Do the different components of the study adhere to the quality criteria of each tradition of the methods involved?***		x	

^*^a. Very small samples compared to target population; b. Discrepancies in some features (gender, ethnicity, social conditions) between participants and target population. **Only some descriptive statistics provided by the authors. ***Some limitations (representativeness of the sample and completeness of statistics) of quantitative research.

**Table 3:** Quality appraisal (Methodological index for non-randomized studies) of the non-controlled study included in the review (31).

1. A clearly stated aim	2
2. Inclusion of consecutive patients	2
3. Prospective collection of data	2
4. Endpoints appropriate to the aim of the study	2
5. Unbiased assessment of the study endpoint	2
6. Follow-up period appropriate to the aim of the study	2
7. Loss to follow up less than 5%	1
8. Prospective calculation of the study size	1

**Table 4:** Quality appraisal (CASP Qualitative Studies Checklist) of the qualitative study included in the review (25).

	Yes	No	?
*Section A: are the results valid?*			
1. Was there a clear statement of the aims of the research?	x		
2. Is a qualitative methodology appropriate?	x		
3. Was the research design appropriate to address the aims of the research?	x		
4. Was the recruitment strategy appropriate to the aims of the research?			x
5. Was the data collected in a way that addressed the research issue?	x		
6. Has the relationship between researcher and participants been adequately considered?			x
*Section B: what are the results?*			
7. Have ethical issues been taken into consideration?	x		
8. Was the data analysis sufficiently rigorous?	x		
9. Is there a clear statement of findings?	x		

## 4 Discussion

Although older persons are considered the most exposed to risks of climate (14), the present review confirms that very little research has been conducted so far about the best way of communicating with these subjects about this matter (28). The bibliographic search of databases of this review, since the first run, showed a wide inequality between all-age and older persons-only records in terms of numbers. Even analysing subpopulations of the all-age studies, the total percentage of older persons was often lower than the other subgroups’ ones thus excluding them from the review.

Communication campaigns on climate change are often directed towards younger demographic groups, likely because they are considered more susceptible to climate change and more open to environmental messages (38). Considering climate change as a problem primarily affecting future generations could thus contribute to a lack of urgency in understanding and studying how to effectively communicate climate change to the older persons (39). Political and economic factors could also interfere in the development of this area of research (37). If policies or funding focus on other aspects of environmental research or specific demographic groups, studies on climate change communication to the older persons may be overlooked.

However, it cannot be ruled out that the low involvement of older adult subjects in such research is also due, at least in part, to a lack of interest among this population to participate in such initiatives. This seems to be confirmed by the study of Rhoades et al. (32), where the meetings were open to all older adults of the community, comprising about 16,000 individuals, but, despite extensive outreach through the city’s three senior centres to encourage large attendance, only 37 older adults were involved. On the other hand, the finding of Moser (37) suggest that older people, when involved, are even more responsive than younger individuals to educational interventions, demonstrating a greater increase in their level of concern about climate change and in their willingness to take a more active role in countering it.

Effective communication with older individuals is crucial for addressing social issues. Current literature emphasizess tailoring communication strategies to specific contexts, establishing mutual understanding, and employing age-appropriate techniques (40–42). Personalized approaches and the use of online tools are also advocated (43, 44). Understanding communication barriers and customizing interactions to meet individual needs are highlighted as significant (45, 46). Additionally, insights into the social and psychological aspects of communication and aging, including languag”s role in shaping identities, are provided (47).

Acknowledging and informing older adults about how climate change affects their health could help reduce harm and ease burdens on this group. It involves adapting personal behaviors to mitigate climate change, actively engaging in societal efforts to address climate issues, and understanding methods to minimize pollution, waste, and conserve energy within healthcare practices or systems (48).

Yet communicating the climate issue could also benefit older people themselves as well as future generations. First of all, awareness of potential risks for their health could led to a more self-protecting behavior in their daily lives (49). Moreover, through involvement in pro-environmental activities, older adults could experience improvements in both physical and mental health. In a survey on 2,032 respondents, about ¼ of whom were people over the age of 65, it was found that volunteering in environmental issues was associated with a 2.6-fold increase in the likelihood of meeting physical activity recommendations (50).

Another aspect of this same issue was the uncertainty about the definition of older persons. In this study we applied the WHO definition that states as an older person a subject aged 65 years and older (7). However, some studies resulting from the search have been excluded because of the age range they choose to adopt to define this population (51, 52).

The complexity of planning a study showing the effects of a communication intervention is demonstrated by the large number of studies discarded at the selection stage for this reason (n = 84). In fact, although some of the excluded articles reported qualitative investigations on the topic, none of them went into the depths of intervention strategies or reported measures of effect. This highlights a lack in study planning, which often does not follow a scientific methodological approach, and suggests a difficulty in engaging older people. In contrast, the results of this review highlight that the most effective climate change communication strategies to the older adults start with their involvement, which appears to be crucial in improving awareness.

The challenges in planning intervention studies on this topic and with this type of population are likely a cause of the lack of studies reporting behavioral change as an outcome. Indeed, observing this type of variable would require interventional study protocols with the enrolment of large samples or cohort studies, both involving extended observation periods. However, the finding reported by Moser (37) that a higher proportion of older subjects than younger ones said they were willing to take an active role in the community on the issue, can at least be regarded as a willingness to change behavior.

The three studies resulting from the selection process explore three different communication strategies of climate change to older persons. In Rhoades et al. (32, 33), a process of awareness-building through meetings and surveys led to a set of recommendations resulting from the participant” concerns and priorities. A key point of this mixed-method case series is the involvement of older persons in every step of the study thus improving their engagement in the topic of climate change making them feel like actors of their own resilience-building process. Involvement is a major feature also in Latter (28) that emphasizes its role in understanding the core values of the older generation. These values would represent a powerful feature to address climate change issues to this population. Both studies show that, through involvement in climate change-related activities, the needs for greater awareness of the issue but, more importantly, for social integration and participation in community life can be met (14).

In Moser (53) a different approach has been pointed out to raise awareness about the climate change topic. The opportunity to visualize the potential impacts of climate change directly on site by the adopted technology is a very strong feature of this project. The OWL technology was used to promote immediate awareness about what the future could reserve for their own life habitat. As demonstrated by results, in the older age groups the level of concern increased as did the willingness to be engaged in community actions about adaptation. Accessibility of this device is a potential answer to the issue of lack of self-confidence of older persons in their expertise or knowledge about environmental issues as well as to their limited e-literacy and confidence in technology adoption (14).

Due to the different nature of the included studies, it is difficult to properly estimate an overall external validity of the results. Qualitative and mixed-methods studies are most often framed in a social constructionist perspective, i.e., based on the needs of the people involved and the characteristics of the local culture, an (53), allowing analytical rather than statistical generalization. However in Rhoades et al. (32) the research was conducted by examining the issues within the participants’ context, acknowledging the various perspectives of thought in a vulnerable group, and fostering action to address these issues to the community.

All the included studies, though with different study design, are of a moderate/high quality. However, none of them reports quantitative data about behavioral changes in the observed population. As a further limitation of current evidence, we could not find data exploiting the association between raising in awareness and engagement of these subjects analysed quantitatively. In an article, excluded from this review (52), these aspects have been investigated by means of a randomized controlled trial involving 988 participants in the US. In this study, participants of different ages were randomly exposed to three diverse writing (2 interventional, 1 control) induction tasks on climate change before completing various self-report measures. Scales on pro-environmental behavioral intentions, on legacy motive induction and total amounts of donation to an environmental charity association were provided as objective outcome measures (52). This article was excluded because, although it enrolled also older participants, and presented separate data by age group, it grouped the older people into a group ranging from 53 to 87 years old. Analysing the Supplementary material, we found that the age group we were interested in (>65 years old) accounted for about 10 percent of the total, thus not matching the inclusion criteria. However, a similar strategy could be implemented and strongly encouraged for future studies on the topic thus providing a better quality of evidence in the field.

Although we found only very few studies addressing our topic of investigation, still some suggestions can be driven about which strategies might be effective in promoting awareness about climate change in older persons. First, it can be inferred from the findings of this review that communicating climate change to older persons by involving them in focus groups may help in raising their awareness on this topic, by promoting a discussion on their needs and beliefs, and on what they have experienced in their past, what they experience in the present and what they will legacy to future generations. Some kind of technological support, actually showing how climate changes will affect the older persons’ close environment, as tested by Moser (37), could also be helpful to make the climate change contents more accessible and easily understandable by this type of population helping them to feel the problem real.

In conclusion, this review demonstrates that that the current literature on climate change communication has mostly ignored the older population, thus showing an important gap that should drive further research in this field. Indeed, we found only three studies that addressed this topic. However, despite the paucity of literature this review indicates the potential for different strategies to increase the awareness of older persons about the issue of climate change. Programs are needed to facilitate involvement, making it easier for diverse groups of older persons to understand and act on climate change: our results identify the involvement of older persons in communication processes, the detection of their priorities and the engaging technologies based on real contexts of their daily lives, as approaches deserving to be pursued and further investigated. Additional studies with both qualitative and quantitative designs are needed; in particular, we suggest that future research should:

- conduct qualitative studies to explore older adults’ needs and concerns about climate change, in a variety of social and environmental contexts
- plan the intervention, starting from participants’ needs and concerns.
- investigate the effectiveness of circumscribed interventions through quantitative controlled studies with older participants, both in terms of increased awareness and raised engagement in pro-environmental behaviors

## Data availability statement

The original contributions presented in the study are included in the article/Supplementary material, further inquiries can be directed to the corresponding author.

## Author contributions

SP: Conceptualization, Data curation, Investigation, Methodology, Project administration, Writing – original draft, Writing – review & editing. DL: Conceptualization, Data curation, Investigation, Methodology, Project administration, Writing – original draft, Writing – review & editing. PZ: Conceptualization, Investigation, Methodology, Supervision, Writing – review & editing. CL: Writing – review & editing. GB: Conceptualization, Supervision, Visualization, Writing – review & editing. MB: Conceptualization, Data curation, Investigation, Methodology, Project administration, Supervision, Visualization, Writing – original draft, Writing – review & editing. FC: Conceptualization, Data curation, Funding acquisition, Investigation, Methodology, Project administration, Supervision, Visualization, Writing – original draft, Writing – review & editing.

## Footnotes

^1^ climateaccess.org
