# Supplementary material for: How to communicate with older adults about climate change: a systematic review
Source: Front Public Health. 2024 Apr 4;12:1347935. doi: 10.3389/fpubh.2024.1347935 (PMC11025664; doi:10.3389/fpubh.2024.1347935)
Supplement: Supplementary file 1 [file Table_1.DOCX]

Supplementary Material

# Supplementary Data

# We conducted our search in the following electronic databases PsycInfo, EBSCO EduSource, EBSCO GreenFile, EMBASE, PubMed, WoS, CINHAL, using this search filter:

# ("climate change" OR "global warming" OR "green issue" OR "green* lifestyle*" OR "sustainable living" OR "climate-smart" OR "global environmental threats" OR "climate matter*" OR "natural disasters" OR "nature disasters" OR "pro-environmental behaviour" OR "pro-environmental behavior" OR "environmental friendly behaviour" OR "environmental friendly behavior") AND (communication OR education OR teaching) AND ("old* age*" OR elder* OR "old* people" OR "65+" OR "over65" OR "old adult*" OR "older adult*" OR "old people" OR "elder*" OR "senior" OR "senium" OR "retiree*" OR "pensioner*" OR "baby boomer*") AND (chang* OR promot* OR influenc* OR support*)

# This filter has been adapted to run on Scopus database in order to better match the inclusion/exclusion criteria. The actual Scopus research strategy was:

# TITLE ( "climate change" OR "global warming" OR "green issue" OR "green* lifestyle*" OR "sustainable living" OR "climate-smart" OR "global environmental threats" OR "climate matter*" OR "natural disasters" OR "nature disasters" OR "pro-environmental behaviour" OR "pro-environmental behavior" OR "environmental friendly behaviour" OR "environmental friendly behavior") AND ( communication OR education OR teaching ) AND ( "old* age*" OR elder* OR "old* people" OR "65+" OR "over65" OR "old adult*" OR "older adult*" OR "old people" OR "elder*" OR "senior" OR "senium" OR "retiree*" OR "pensioner*" OR "baby boomer*" ) AND ( chang* OR promot* OR influenc* OR support* ) AND ( LIMIT-TO ( SUBJAREA , "ENVI" ) OR LIMIT-TO ( SUBJAREA , "SOCI" ) OR LIMIT-TO ( SUBJAREA , "MEDI" ) OR LIMIT-TO ( SUBJAREA , "PSYC" ) OR LIMIT-TO ( SUBJAREA , "NEUR" ) OR LIMIT-TO ( SUBJAREA , "HEAL" ) ) AND ( EXCLUDE (DOCTYPE , "ch" ) OR EXCLUDE ( DOCTYPE , "bk" ) OR EXCLUDE ( DOCTYPE , "cp" ) OR EXCLUDE ( DOCTYPE , "no" ) OR EXCLUDE ( DOCTYPE , "cr" ) OR EXCLUDE ( DOCTYPE ,"sh" ) ) AND ( EXCLUDE ( EXACTKEYWORD , "United States" ) OR EXCLUDE ( EXACTKEYWORD , "China" ) OR EXCLUDE ( EXACTKEYWORD , "Nonhuman" ) OR EXCLUDE ( EXACTKEYWORD , "Animals" ) OR EXCLUDE ( EXACTKEYWORD , "Animal" ) OR EXCLUDE (EXACTKEYWORD , "Australia" ) OR EXCLUDE ( EXACTKEYWORD , "Europe" ) OR EXCLUDE (EXACTKEYWORD , "Canada" ) OR EXCLUDE ( EXACTKEYWORD , "Forestry" ) OR EXCLUDE (EXACTKEYWORD , "United Kingdom" ) OR EXCLUDE ( EXACTKEYWORD , "India" ) OR EXCLUDE ( EXACTKEYWORD , "Africa" ) OR EXCLUDE ( EXACTKEYWORD , "Cultivation" ) )
